# Approximate CT-free patient specific attenuation correction method for cardiac-dedicated pinhole CZT system: a feasibility phantom study

**DOI:** 10.1186/s40658-026-00893-1

**Published:** 2026-05-17

**Authors:** Michel Hesse, Florian Dupont, Véronique Roelants

**Affiliations:** Nuclear Medicine Department, Cliniques Universitaires Saint-Luc, 10 Avenue Hippocrate, 1200 Brussels, Belgium

**Keywords:** MPI, CZT, Pinhole, Cardiac, Attenuation, SPECT/CT

## Abstract

**Background:**

Multi-pinhole cardiac-dedicated CZT gamma cameras improve clinical workflow by reducing acquisition duration and/or injected activities. As most of these systems lack integrated CT scanner, attenuation artifacts may appear in the reconstructed image depending on patient morphology. External CT-based attenuation correction is possible but requires manual SPECT-CT registration and increases patient absorbed dose. The aim of the present study was to develop an approach to estimate patient-specific attenuation maps directly from emission data acquired on a pinhole cardiac CZT camera.

**Methods:**

Two anthropomorphic torso phantoms were used. Their heart wall (with or without defects), liver insert and background cavity were filled with ^99m^Tc solutions to simulate clinical activities. Reference acquisitions were obtained using a CZT SPECT/CT system. For each phantom, two acquisitions were performed on a pinhole cardiac CZT camera: a standard cardiac acquisition and a second acquisition with detectors radially moved away. Combination of both acquisitions enabled delineation of the body contour and segmentation of soft-tissue and lung regions. From this segmentation, a CT-free attenuation map was generated and imported into the manufacturer’s reconstruction software to produce attenuation corrected (AC) images. These CT-free AC images were semi-quantitatively compared, using 17-segment polar maps, to (1) images corrected using a registered external CT, (2) AC images from the CZT SPECT/CT system, and (3) the true activity distribution in the phantoms. A three-dimensional voxel-wise comparison was also performed between CT-free AC and CT-based AC images of the pinhole CZT camera.

**Results:**

CT-free attenuation maps produced tracer distribution and 17-segment polar maps closely matching those obtained with CT-based attenuation correction. Heart wall defects in the anterior and inferior areas remained clearly visible except for half-obstructive defects. Differences relative to CZT SPECT/CT images were mainly attributable to camera geometry and reconstruction algorithm.

**Conclusions:**

The novel CT-free patient-specific attenuation correction method yields images comparable to CT-based AC images and shows good agreement with AC images from a CZT SPECT/CT system. This approach could enhance the diagnostic performance of current cardiac pinhole CZT cameras without requiring additional CT acquisitions.

**Supplementary Information:**

The online version contains supplementary material available at 10.1186/s40658-026-00893-1.

## Introduction

Pinhole cardiac-dedicated CZT cameras have been commercially available for over a decade now [1]. These systems improve cardiac studies workflow by significantly reducing acquisition duration and/or injected activities compared to conventional gamma cameras [2]. Most of these systems do not include a CT scanner to maintain affordability and a small footprint, and therefore cannot account for gamma-ray attenuation by patient tissues. However, it is widely acknowledged that attenuation correction improves the diagnostic accuracy of myocardial perfusion (MP) SPECT [3–7].

In such camera, attenuation correction can be achieved using an external CT scan of the patient’s thorax, but this approach requires manual registration with the SPECT data and increases the patient’s absorbed dose [8]. Alternatively, reconstruction methods have been developed to generate attenuation maps solely from SPECT emission data [9–11]. Collecting and processing scatter data may help in segmenting different tissues to generate attenuation maps [10, 12]. However, these approaches are not applicable to cardiac-dedicated pinhole CZT cameras due to their limited field of view (FOV) [13]. These cameras focus on a small volume around the patient’s heart, resulting in a lack of qualitative data outside the FOV, which is essential for generating an accurate attenuation map.

With the recent emergence of artificial intelligence (AI), attenuation correction approaches based on deep learning (DL) have been developed specifically for MP SPECT [14–24]. These interesting techniques enable the generation of attenuation corrected (AC) images only from emission scan data. However, they require large training datasets acquired using a similar camera with a CT scanner [17]. Training the AI model on AC images obtained from a different SPECT camera may result in artifacts in the reconstructed images due to differences in detector properties or camera geometry [25].

In this study we present a novel method to estimate the patient attenuation map only from emission scans obtained on a cardiac-dedicated pinhole CZT camera (pinhole CZT) without the use of an external CT scanner. The method was evaluated with cardiac phantoms acquired sequentially on a pinhole CZT and on a CZT SPECT/CT system. CT-free AC images from the pinhole CZT were compared head-to-head with (1) AC images generated using an external CT, (2) AC images from the CZT SPECT/CT system, and (3) the ground-truth activity distribution derived from the known phantom activity concentration.

## Materials and methods

### Phantoms

A first anthropomorphic torso phantom (TP) (DataSpectrum, Durham, NC) was used with cardiac, liver and lung inserts. Three configurations were evaluated:

1. TPN: No defect in the heart wall;
2. TPW: One defect in the infero-basal segment (90° ⋅ L ⋅ W) and one defect in the mid-anterior segment (45° ⋅ L ⋅ W);
3. TPW2: One defect in the infero-basal segment (90° ⋅ L ⋅ W) and one defect in the mid-anterior segment (45° ⋅ L ⋅ W/2).

All defects had a length L of 20 mm along the heart long axis, and W corresponds to the heart wall thickness of about 10.3 mm. All defects were water filled except for the 45° ⋅ L ⋅ W/2 defect which was not fillable. Fillable defects contained no activity to simulate fully obstructive defects, while the solid half-width defect represented a partially obstructive defect.

For all 3 TP configurations, the heart wall, liver and body compartments were filled with approximately 58 MBq, 353 MBq and 1090 MBq of ^99m^Tc, respectively. The resulting activity concentration ratios with respect to the heart wall (121 ml) were approximately 0.61 for the liver (1200 ml) and 0.24 for the body compartment (9350 ml), consistent with clinical conditions. The activity injected into the body compartment simulates the patient’s blood pool. The heart cavity and lungs were filled with non-active water, and lung density was reduced by adding polystyrene beads.

A second anthropomorphic Heart/thorax phantom (HTP) (Radiology Support Devices, Inc, Long Beach, CA, USA) was used with a 3D-printed heart insert, without any defect. The left ventricular (LV) wall (190 ml), the liver (980 ml) and the thoracic cavity (8500 ml) were filled with 37.5 MBq, 93.2 MBq and 326 MBq of ^99m^Tc, respectively. These activities were chosen to mimic clinical conditions. To simulate a more corpulent patient, an additional male thorax overlay (Radiology Support Devices, Inc, Long Beach, CA, USA), consisting in a solid polyurethane part that fits the anterior part of the Heart/thorax phantom, was used (see Fig. 3). This configuration is referred to as HTPO in the following.

### Acquisitions

Standard SPECT acquisitions (Std scan) were performed on a Discovery 530c (General Electric Healthcare, Haifa, Israel) (D530c), immediately followed by a second 5 min acquisition with the detectors retracted to allow visualization of the phantom boundaries on the monitoring screen (Far scan). Reference SPECT/CT acquisitions were performed on a GE StarGuide (GE Healthcare, Haifa, Israel), equipped with immovable wide-energy high resolution (WEHR) collimators. SPECT acquisition parameters are summarized in Table 1, along with the total counts recorded in the photopeak window for all phantom configurations. Long duration acquisitions were performed for TP configurations to generate high statistics images, while HTP and HTPO configurations were acquired using clinical acquisition durations to reproduce count statistics representative of routine MP SPECT. In total 6 acquisitions were performed with the TP and 4 with the HTP.

**Table 1:** SPECT acquisition parameters

	TP configurations		HTP configurations	
	Discovery 530c	StarGuide	Discovery 530c	StarGuide
Number of projections	TPN: 19 TPW: 19 TPW2: 19	TPN: 3379 TPW: 3411 TPW2: 3403	HTP: 19 HTPO: 19	HTP: 3376 HTPO: 3491
Total duration (min)	30 + 5	45	5 + 5	12
Matrix size	32 ⋅ 32	16 ⋅ 112	32 ⋅ 32	16 ⋅ 112
Pixel size (mm)	2.46	2.46	2.46	2.46
Photopeak window (keV)	140.5 ± 10%	140.5 ± 10%	140.5 ± 10%	140.5 ± 10%
Scatter window (keV)	120 ± 5%	120 ± 5%		
Total acquired counts in photopeak window (cnts)	TPN: 189,126,110 TPW: 125,997,365 TPW2: 167,986,035	TPN: 279,832,002 TPW: 262,777,867 TPW2: 244,303,581	HTP: 6,119,504 HTPO: 3,803,103	HTP: 10,563,349 HTPO: 8,035,308

Table 1: Acquisition parameters for the 2 cameras and for the five phantom configurations. For the Discovery 530c, the total duration is split into the duration of the standard heart acquisition plus the duration of the Far scan. For the StarGuide, the number of projections is automatically determined by the system based on the phantom position on the camera bed. TP: torso phantom; HTP: heart/thorax phantom; N: no defect; W: with defects; O: with thorax overlay.

### CT-free attenuation map generation

**Fig. 1:**
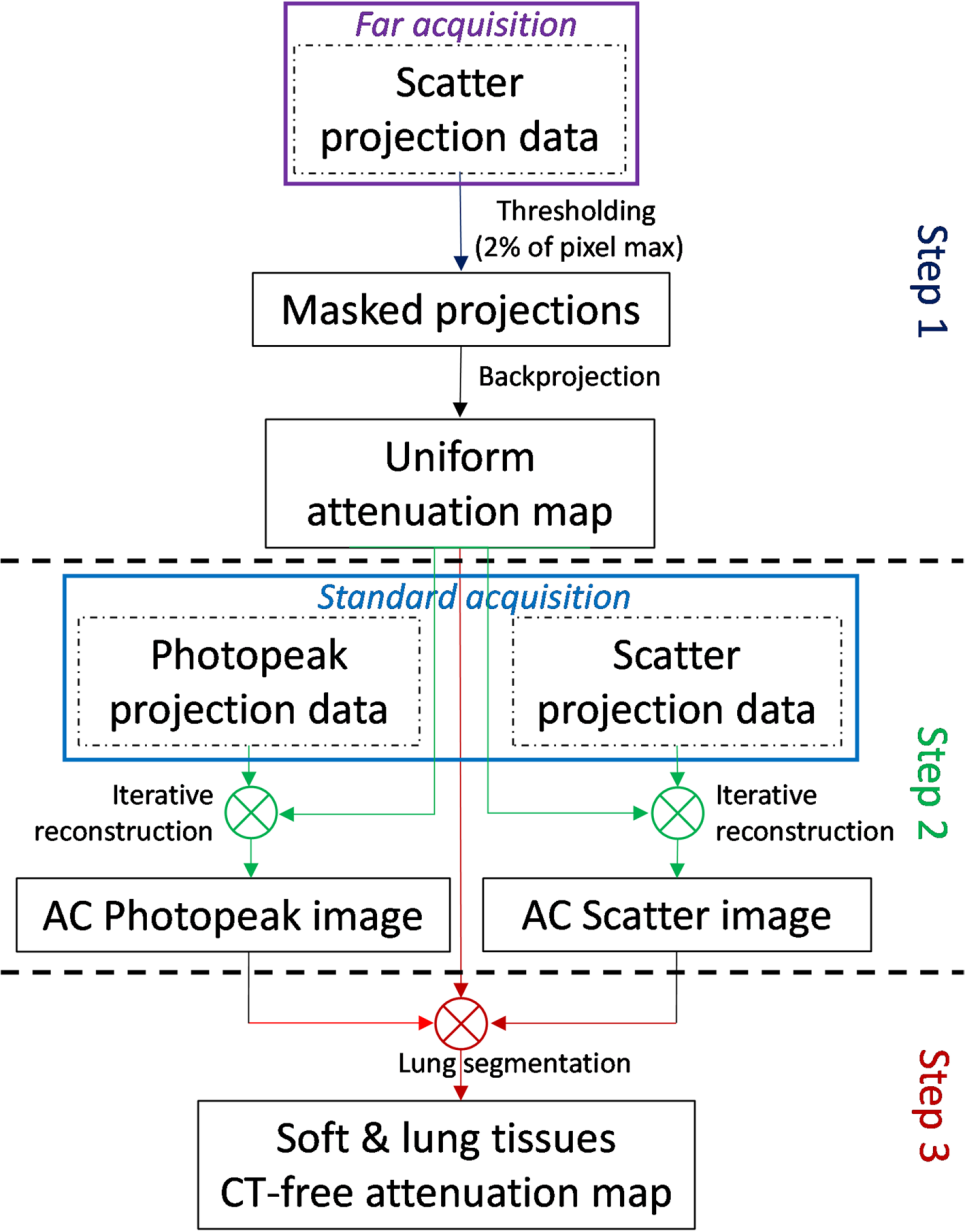
Flowchart of the novel CT-free attenuation map generation

The algorithm used to generate the proposed CT-free attenuation map consists of three successive steps as illustrated in Fig. 1. A detailed description of steps one and three of the algorithm is provided in the Supplementary material.

Figure 1 Flowchart of the novel CT-free attenuation map generation method based only on the Standard and Far scatter acquisitions data. AC, attenuation corrected.

In the first step, scatter projections acquired during the Far scan are thresholded at 2% of the maximum pixel value to remove background noise. The resulting masked projections are backprojected into image space to obtain a body volume, which is then converted into a uniform attenuation map by assigning a fixed attenuation coefficient of 0.12 cm^− 1^ to all voxels. This value corresponds to the attenuation coefficient of water and accounts for the contribution of scattered photons within the energy window [26].

In the second step, this uniform attenuation map is used to reconstruct AC activity distributions of the Std scan for both the photopeak and scatter energy windows, using 10 iterations of the maximum-likelihood expectation-maximization (MLEM) algorithm.

In the third step, the AC Photopeak and AC Scatter images are jointly processed to identify voxels corresponding to the lung tissue. First, the smallest ellipsoid enclosing the heart in the AC Photopeak reconstruction is computed using the Khachiyan algorithm [27], based on heart boundaries detected with a gradient-based approach (see Supplementary material). Compared with threshold-based methods, this ellipsoid approach is less sensitive to potential myocardial perfusion defects. In the slices containing the heart, the lung region is segmented from the left boundary of the ellipsoid using the AC Scatter image. The lung segmentation is then propagated toward superior slices (i.e., toward the shoulders) by adapting the lung contour from the previous slice according to local image gradients. The resulting lung region is finally incorporated into the uniform attenuation map to generate a two-tissue CT-free attenuation map. Attenuation coefficients for soft tissue and lung were set to values measured on the phantom CT, namely 0.15 and 0.053 cm^− 1^, respectively.

The CT-free attenuation map generation algorithm is fully automatic and requires only the Std and Far projections as inputs, along with the corresponding detector radii, which are used to align the reconstruction spaces of both acquisitions. The complete processing pipeline requires approximately 5 min on a standard Intel Core i7 laptop without GPU acceleration, with the MLEM reconstructions constituting the most time-consuming step.

### Reconstructions

SPECT acquisitions were reconstructed with the manufacturer iterative algorithm specific to each camera (reconstruction parameters are summarized in Table 2). The TP images were corrected for scatter by using the double energy window method (scatter window setting in Table 1). For the StarGuide data, the standard scatter factor developed for NaI detectors (1.17 for the energy windows defined in Table 1) was used, as recommended by the manufacturer. For the D530c system, an empirical scatter factor of 0.7 was derived by fitting the energy spectra of each pinhole-detector pair with a Gaussian photopeak including a low-energy tail and a sigmoid-like Compton scatter component.

**Fig. 2:**
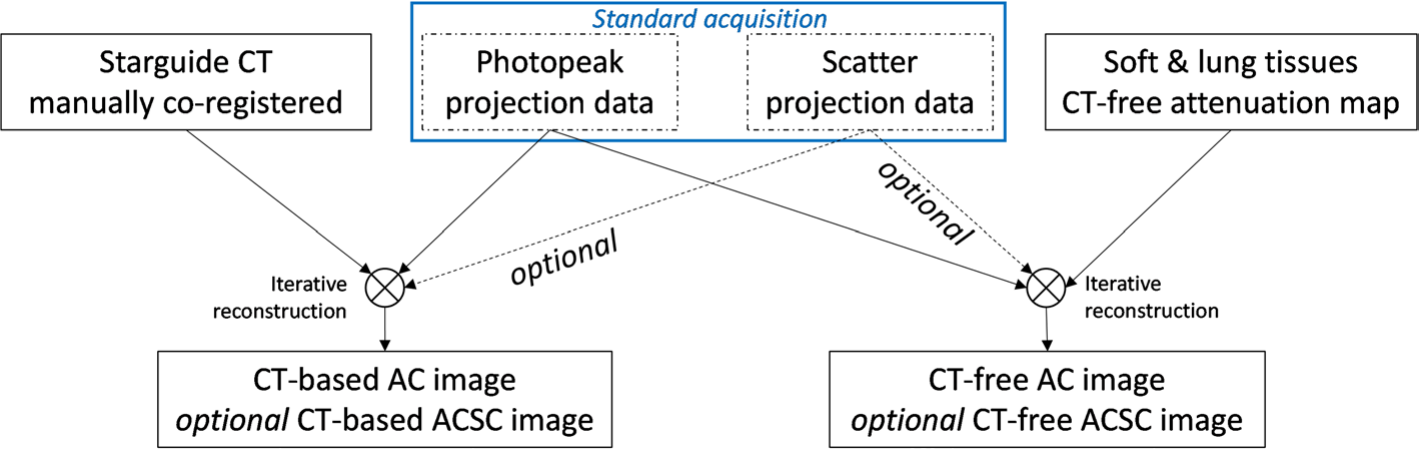
Flowchart of the reconstructions of the D530c acquisitions

No scatter correction was applied on the HTP because of its degrading effect on the image quality due to the lower statistics. D530c CT-free AC images were generated by incorporating the attenuation maps produced by our novel CT-free method into the reconstruction algorithm. For comparison, CT-based AC images were reconstructed using the semi-automatically registered StarGuide CT (Xeleris software, GE Healthcare, Haifa, Israel) (see Fig. 2). For the TP, the number of iterations was optimized to achieve quantitative convergence in the reconstructed images, as the statistical noise was limited by the high injected activities and long acquisition times. For the HTP, the numbers of iterations recommended by the camera manufacturer, and providing convergence for clinical acquisition conditions, were applied.

Figure 2 Flowchart of the iterative reconstructions of the D530c acquisitions with attenuation correction using the external Starguide CT or the novel CT-free attenuation map. Scatter corrected images (ACSC) were generated for the TP configurations while only AC images were reconstructed for the HTP configurations. AC, attenuation corrected; SC, scatter corrected.

As the ^99m^Tc activity injected into the different phantom compartments was known, a ground-truth activity image could be generated for each phantom. An activity value was assigned to each voxel according to its anatomical location on the StarGuide CT image. The resulting true image was then smoothed using a 15 mm-FWHM Gaussian filter to approximate the cameras spatial resolution, and subsequently resampled to match the reconstruction voxel size of each camera (Table 2). This ground-truth image served as the reference for evaluating the reconstructed images obtained on both cameras.

The reconstructed images were semi-quantitatively evaluated using the American Heart Association (AHA) 17-segment polar map model. The mean relative activity in each segment was computed using the Quantitative Perfusion SPECT software (QPS, Cedars-Sinai Medical Center). The percentage ratio of the mean relative activity was calculated between each reconstruction and the ground-truth image, as well as between the D530c CT-free and CT-based AC images. In addition, a three-dimensional voxel-wise comparison was performed between CT-free and CT-based AC reconstructions of the D530c acquisitions for the voxels of the cardiac insert.

**Table 2:** SPECT reconstruction parameters

Iterative algorithm	Discovery 530c	StarGuide
	MLEM	Q.Clear
Iteration number	TP: 250 HTP: 60	TP: 250 HTP: 50
Iteration subset	/	10
Regularization	OSL Green (α = 0.7; β = 0.3)	MRP (β = 0.4; “By Sens” option active)
Post-filter	Butterworth (cutoff = 0.37; order = 7)	Gaussian 15 mm
Voxel size (mm)	4	4.92

Table 2: Iterative algorithm and parameters used to reconstruct the SPECT acquisitions performed on the Discovery 530c and StarGuide cameras. The StarGuide “By Sens” option is made available by the manufacturer to incorporate information about the acquisition’s geometry into the reconstruction.

## Results

### CT-free attenuation map

**Fig. 3:**
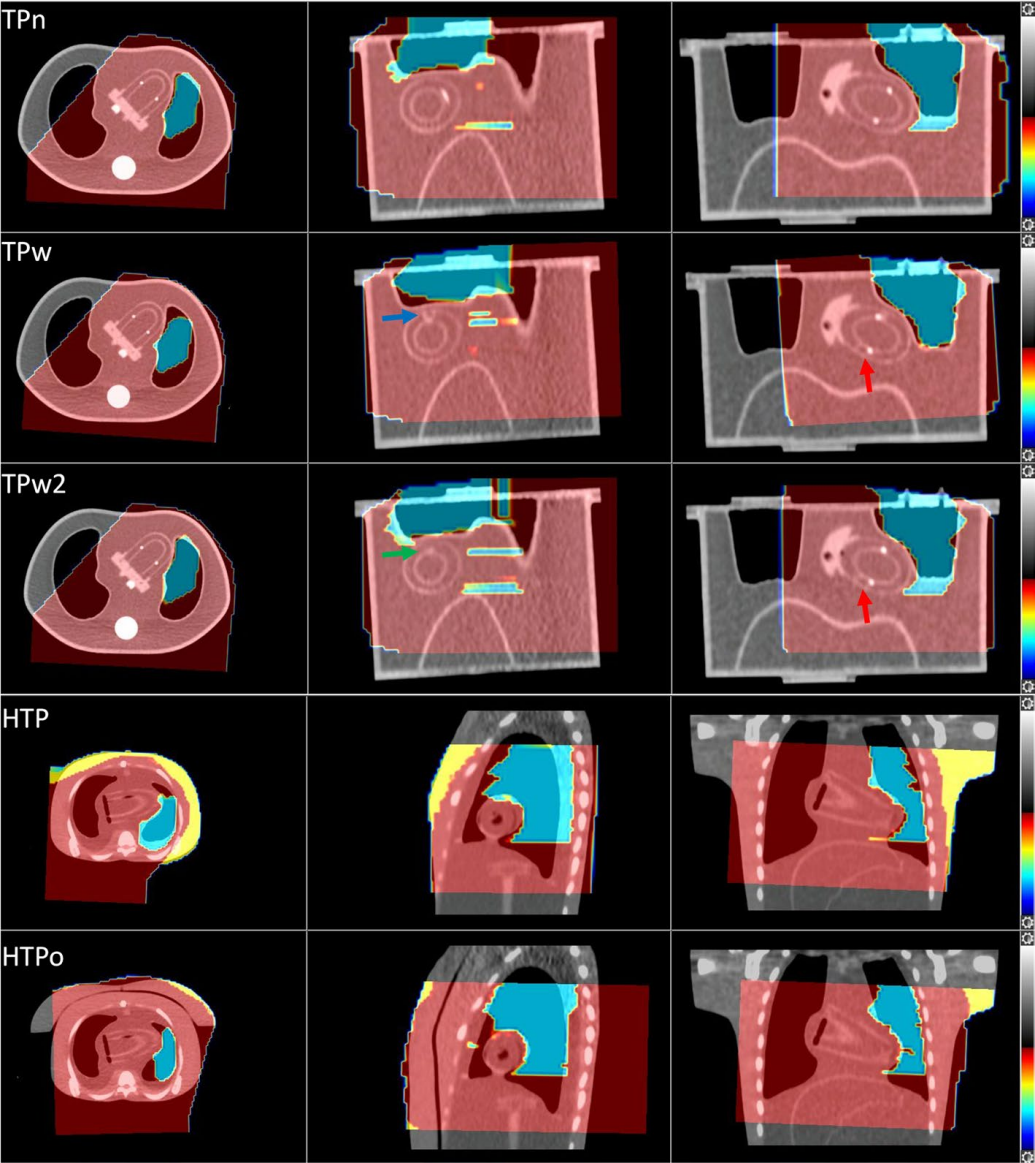
Comparison of CT-free attenuation maps with CT scans for all phantom configurations

Figure 3 shows the generated CT-free attenuation map (blue-red color scale) for each TP and HTP configuration, fused with the corresponding StarGuide CT image (grayscale) (see Supplementary Material for the co-registration details). All anatomical features of the anthropomorphic phantoms are clearly visible on the CT image: liver, lungs and heart as well as the cylindrical spine insert for the TP configurations and the rib cage for the HTP configurations. Red, blue and green arrows indicate the heart wall defects in TPW and TPW2.

As parts of the HTP (beyond the ribs) are made of solid polyurethane, they contain no activity and are therefore not clearly visible in the detector projections. Depending on the activity distribution and the relative detector position, these polyurethane regions can be partially visible because they may scatter the incoming gammas towards the detector pinholes. To avoid introducing bias, these non-active and poorly detected regions were manually added to the attenuation map using the StarGuide CT. They appear in yellow in Fig. 3 for the HTP and HTPO configurations.

By design, the CT-free attenuation map exhibits straight cut edges on the left and the inferior sides, because the D530c detectors see the patient only from above and from the right. Tissues far below and far on the right of the heart play no role in the attenuation of the detected photons coming from the heart but may of course affect the image reconstruction as they are seen, from far away, by some of the D530c detectors.

Figure 3 3D view of the generated CT-free attenuation maps (in blue-red color scale) fused with the phantoms CT from the StarGuide (in grayscale). Soft and lung tissues on the generated CT-free attenuation map appear in red and light blue, respectively. Yellow areas on HTPs were manually added to the attenuation map, from the corresponding CT, to include the phantom borders that are not seen by the detectors because they are free of activity.

### Two-dimensional polar map analysis of AC reconstructions and ground-truth for all phantom configurations

**Fig. 4:**
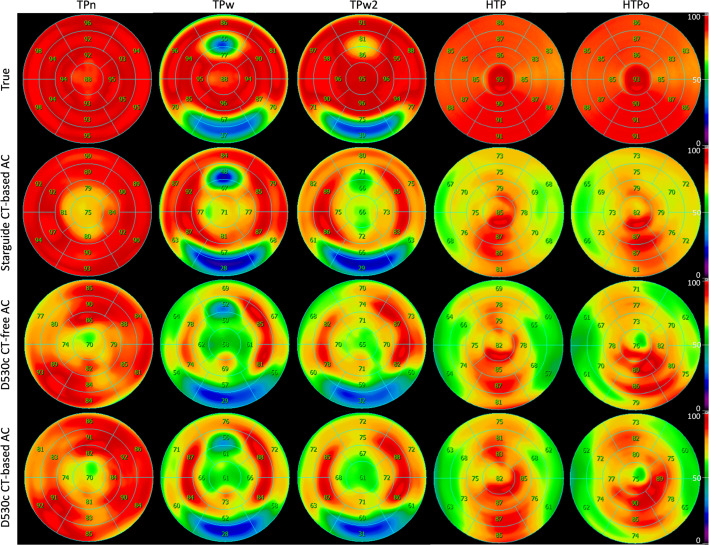
Polar maps of ground-truth image, CT-based and CT-free AC images of all phantoms

Figure 4 illustrates the comparison of the TP and HTP AC images using the AHA 17-segment model polar maps [28]. This representation clearly demonstrates a homogeneous activity distribution within the heart wall of the ground-truth images for both the TPN and HTP configurations. In contrast, the activity distribution appears more heterogeneous in all reconstructed AC images, particularly for the HTP configurations. Nevertheless, the CT-free AC polar maps show good agreement with those obtained using CT-based attenuation correction. Defects spanning the whole heart wall thickness are well visible on all reconstructions of TPw and TPw2, while the partially obstructive defect in TPw2 is only observed in the StarGuide image, thanks to the better spatial resolution of this camera.

Figure 4 American Heart Association (AHA) 17-segment model polar maps of the TP and HTP images, reconstructed with scatter and attenuation corrections for the TPs, and only attenuation correction for the HTPs. The ground-truth image, the StarGuide CT-based AC, the D530c CT-free AC and the D530c CT-based AC reconstructions are displayed on lines 1 to 4, respectively.

**Fig. 5:**
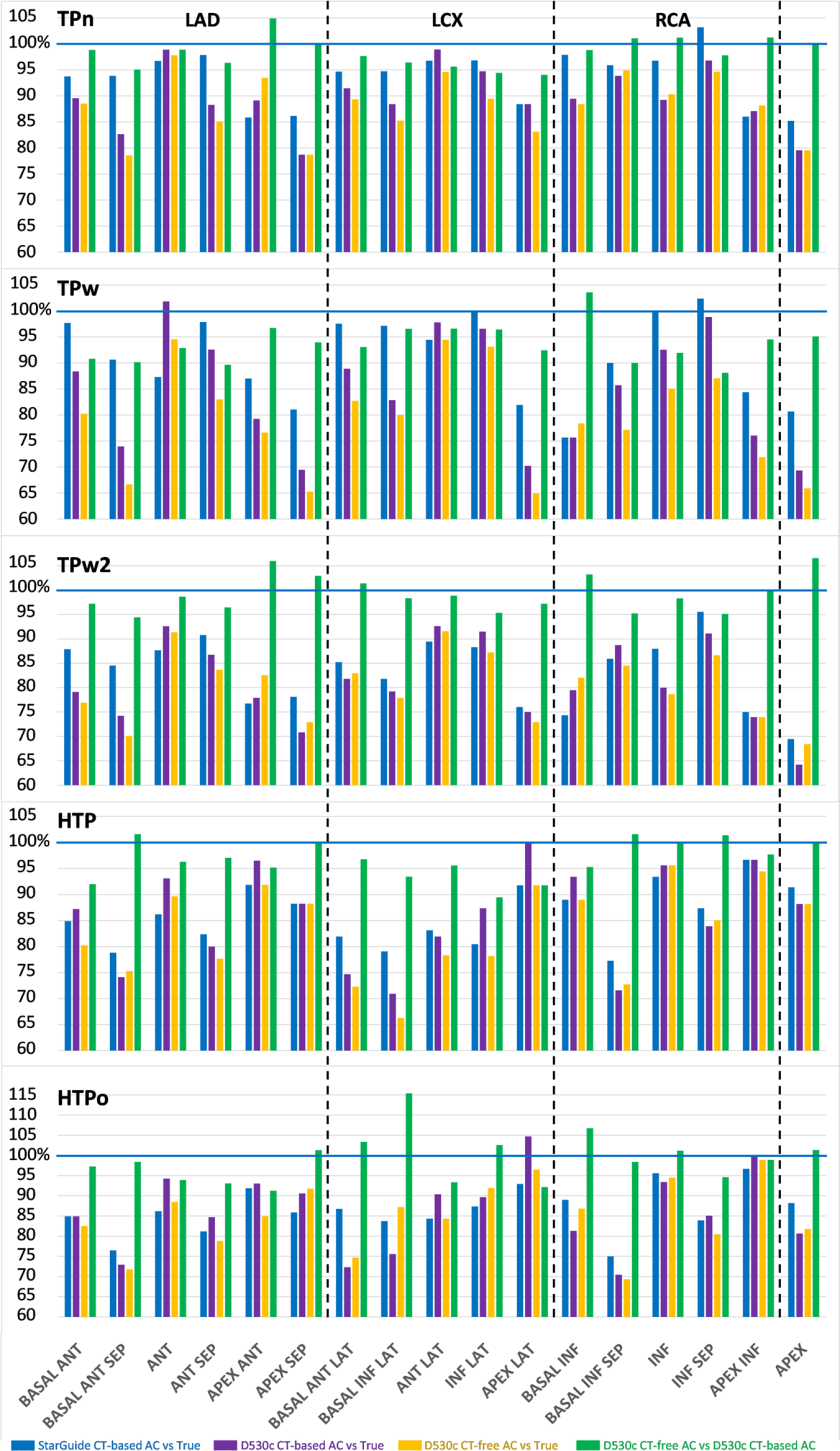
Relative mean activity of the 17 segments of the polar maps for CT-based and CT-free AC images

Figure 5 Relative mean activity of the 17 segments of the American Heart Association (AHA) polar maps for the TP and HTP SPECT images from Fig. 4. The horizontal blue lines correspond to the perfect match of the segment activity between two images. The blue, purple, and yellow bars represent the mean activities-relative to the ground-truth-obtained using the Starguide CT-based attenuation correction, D530c CT-based attenuation correction, and D530c CT-free attenuation correction, respectively. The green bar represents the comparison between CT-free and CT-based AC values for the D530C acquisitions. Segments are identified according to their location: basal (BASAL), septal (SEP), inferior (INF), anterior (ANT), lateral (LAT), apex (APEX); and grouped according to the feeding artery: left anterior descending (LAD), left circumflex (LCX) and right coronary (RCA).

Relative quantification of the segments’ activity is illustrated in Fig. 5 for the 5 phantom configurations. Regarding the TP, for the StarGuide camera, the true activity of TPN is retrieved with an error of less than 10%, except in the apical segments that display a drop of activity. Similar differences are obtained for TPW in the segments not covered by the defects. Larger differences, up to 15%, are observed for TPW2. Regarding the D530c reconstructions, up to 20% and 30% errors, in the basal and apical segments, are obtained for both AC images compared to the true image for TPW and TPW2, respectively. However, activity differences of less than 10% are obtained between the CT-based AC and CT-free AC reconstructions of the D530c for the 3 TP configurations. On the other hand, the recovery of the heart wall activity in HTP and HTPO with the StarGuide is less good, with an error, relatively to the true activity, of less than 15% for all segments but the most basal ones for which the error increases up to 25%. Similar trends are observed on the D530c, with errors up to 20% and 15% for all the segments but the basal ones, for the D530c reconstructions with the CT-free method and with the StarGuide CT, respectively. For the basal segments the error increases up to 30% in both cases. Again, activity differences of less than 10% are obtained between the CT-based AC and CT-free AC reconstructions of the D530c for both HTP configurations, except for the latero-basal segment on the HTPO where the error reaches 15%.

### Three-dimensional voxel-wise analysis of CT-free AC and CT-based AC reconstructed images

**Fig. 6:**
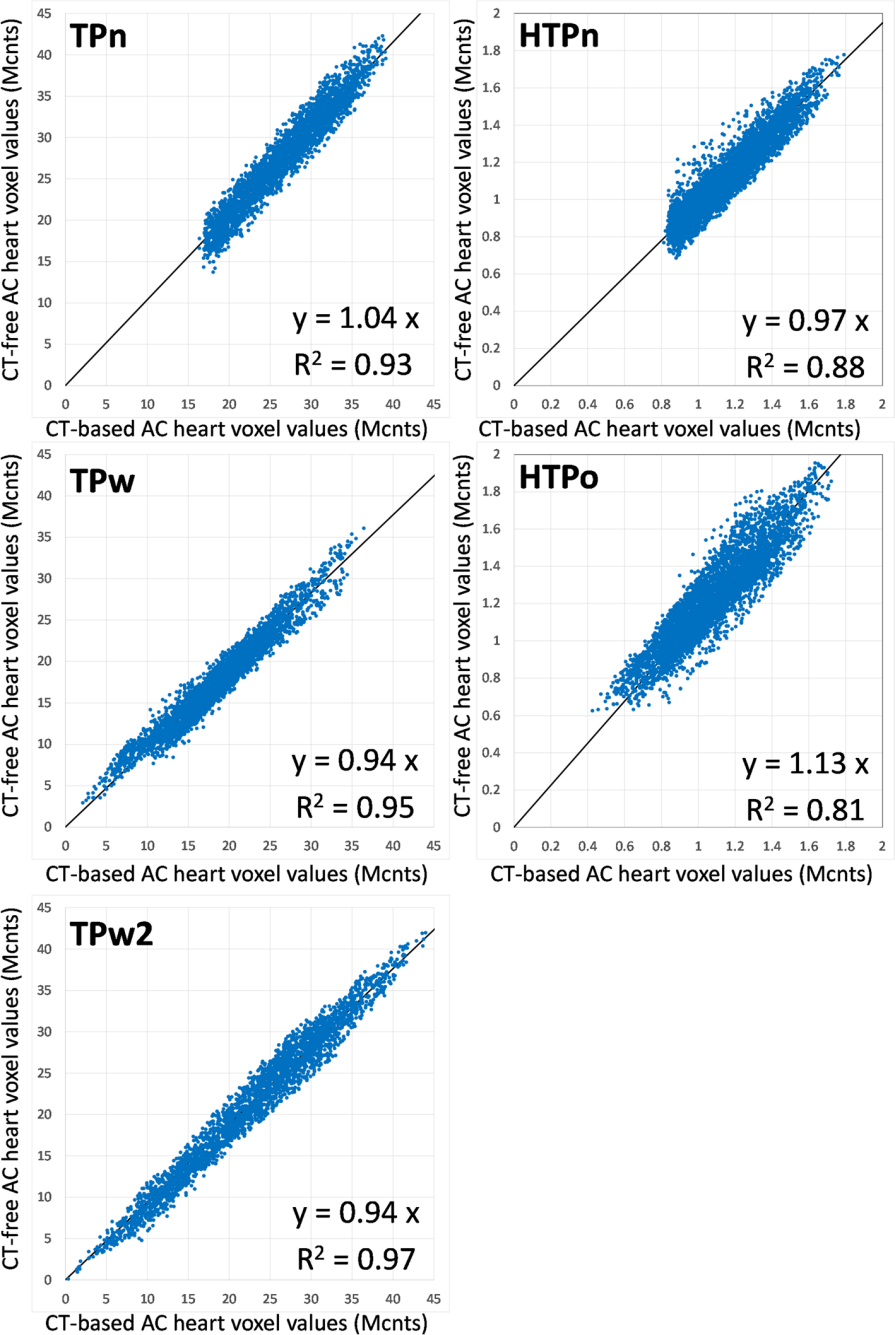
Voxel-wise correlation between CT-based AC and CT-free AC images of the D530c

The three-dimensional voxel-wise comparison between the CT-based AC and CT-free AC images from the D530c acquisitions is illustrated on Fig. 6 for all image voxels encompassing the cardiac insert. The quality of the quantitative correlation between the 2 images is represented by the Pearson correlation coefficient R^2^, which is above 0.93 and 0.81 for the TP and HTP phantom, respectively.

Figure 6 Voxel-wise correlation between CT-based AC and CT-free AC images of the D530c for the 5 phantom acquisitions. Each dot corresponds to an image voxel associated to the cardiac insert. The Pearson correlation coefficient R^2^ and the linear regression equation are given for each phantom.

## Discussion

The most salient finding of this phantom study is that the novel CT-free patient specific attenuation map yields images that closely match those obtained with CT-based attenuation correction, and show good agreement with AC CZT SPECT/CT images. These results were obtained using phantoms under both high-counting-statistics conditions and counting statistics comparable to those encountered in clinical studies.

Despite their limited spatial resolution, the CT-free attenuation maps reasonably reproduced the soft tissue and lung regions of both phantom geometries (Fig. 3). Artifactual lung-like regions were observed in TPs, particularly near the boundaries of the D530c FOV and in posterior areas. These artifacts are primarily attributable to reconstruction effects related to the pinhole geometry and incomplete volume coverage. However, their impact on myocardial quantification was limited, as shown by polar map analysis and voxel-wise correlations (Figs. 4, 5, 6).

In the HTP configurations, some solid polyurethane components of the phantom (shown in yellow in Fig. 3) were manually incorporated into the attenuation map to avoid introducing bias in the final reconstructions (see Fig. S6, Supplementary Material, for a comparison of reconstructed images with and without the manual edition of the attenuation map). Fewer manual adjustments were required for the HTPO configuration than for the standard HTP configuration, likely owing to differences in the relative positioning of the phantom and the detectors during acquisition. Such artifacts are not expected under clinical conditions, as blood pool activity generally ensures sufficient tracer uptake throughout most soft tissues. Even tissues with negligible activity, such as adipose tissue, remain visible owing to scattered gamma photons originating from adjacent active regions.

Comparison between D530c CT-free AC and CT-based AC images revealed no major discrepancies across all phantom experiments (Figs. 4, 5, 6, and Fig. S7–S11 in the Supplementary Material). Only four out of 85 myocardial segments exhibited differences greater than 10% with a maximum observed deviation of 15%. All full-thickness perfusion defects were correctly identified using both the CT-free and CT-based AC methods, while a partially obstructive anterior defect could not be recovered by either approach. Voxel-wise analysis demonstrated a strong linear correlation between CT-free AC and CT-based AC within the cardiac insert. These findings suggest that the novel CT-free method represents a viable alternative to external CT for attenuation correction of D530c images.

All reconstructions of the TPW and TPW2 configurations exhibited reduced activity in the apical region, which may be attributed to suboptimal scatter correction. The standard scatter factor developed for NaI detectors and recommended by the manufacturer may not be optimal for CZT detectors due to the characteristic low-energy tail of their photopeak spectrum [29]. The empirical scatter factor (0.7) derived for the D530c system improves activity recovery in the apical region compared with the standard factor (1.17) (data not shown); however, a residual apical defect remains visible. Although true apical thinning, as observed in clinical patients, cannot be invoked in this phantom study, it should be noted that the cardiac insert used in the TP configuration exhibits manufacturing-related variations in wall thickness at the apex. These structural irregularities may contribute to artifactual apical thinning in the reconstructed images.

For the HTP configuration, pronounced activity deficits were observed in the anterior, septal, and lateral cardiac segments. These deficits are not related to attenuation correction, as they are already present in the non-attenuation-corrected images (Fig. S5, Supplementary material). The origin of these inhomogeneities remains uncertain but may be related to undetected tracer leakage through the walls of the heart insert.

Activity deficits in the lateral and septal regions were more pronounced in the D530c HTP reconstructions but were also observed in TPW and TPW2 configurations. Attenuation-related artifacts in pinhole CZT cameras have been previously reported [30]. Furthermore, the activity distribution in D530c images appeared markedly more heterogeneous than in images acquired using the StarGuide system or in the TPN images. Hindorf et al. [31] demonstrated that optimal centering of the patient’s heart within the FOV is critical for minimizing artifacts when using the D530c CZT camera. Although heart positioning was carefully controlled in this study, perfect alignment, including rotational orientation, could not be guaranteed across all three TPs, partly due to the non-flat geometry of the camera table.

Reconstructions based on StarGuide CT attenuation maps (Fig. 4, line 4) and the D530c NAC images (Fig. S5, Supplementary material) clearly indicate that these artifacts are not attributable to deficiencies in the CT-free attenuation maps generated by our novel method. Rather, the observed activity deficits are related to the intrinsic pinhole geometry of the D530c system, whose sensitivity profile within the reconstruction volume differs substantially from that of parallel-hole collimator systems [4]. Additionally, regions containing activity that are not simultaneously visible to all pinhole-detector pairs can influence the reconstructed activity distribution within the FOV. This effect depends on tracer uptake outside the FOV, such as gastrointestinal activity, as well as on the specific characteristics of the manufacturer’s reconstruction algorithm [32].

Before clinical implementation, the novel method and its associated parameters must be validated using patient data. In particular, soft-tissue and lung attenuation coefficients should be derived from population-based averages. The validity of the two-tissue approximation, which neglects bone attenuation, also requires evaluation in a clinical setting. Although all phantoms in this study included liver activity representative of typical clinical conditions, further investigation is needed to assess the influence of physiological sub-diaphragmatic uptake on the method. Moreover, the present work does not account for patient motion, including cardiac contraction and respiratory motion, whose effects should be evaluated in patient studies. Finally, optimization of the Far scan duration using patient data is necessary to reduce acquisition time and improve patient comfort while maintaining sufficient information for accurate body contour extraction.

An alternative to generating an attenuation map is to directly produce AC images from acquisition data using deep-learning (DL) algorithms [14–24]. While both our method and DL approaches avoid the additional radiation associated with CT scans, DL methods require large training and validation datasets to prevent potential biases. This challenge is particularly relevant for cardiac-dedicated cameras, which often lack SPECT/CT counterparts. For the D530c, the closest equivalent is the Discovery 570c (GE Healthcare, Haifa, Israel), which is no longer commercially available and is installed in only a few centers worldwide.

Deploying DL algorithms in clinical routine is straightforward, as they typically rely on standard acquisition data. In contrast, our CT-free approach requires an additional acquisition with retracted detectors. However, it generates a fully patient-specific attenuation map derived solely from the patient’s own data. While DL methods may incorporate auxiliary inputs such as patient gender [21], they remain dependent on external training datasets.

Recently, Du et al. [23] suggested that DL-based attenuation correction could even outperform CT-based AC by avoiding potential registration errors between NM and CT images. Although our CT-free method also relies on two acquisitions with different detector radii, it does not involve patient repositioning and is therefore less prone to such misregistration errors. Moreover, a key advantage of the proposed approach is that it produces an attenuation map that can be directly inspected, enabling quality control of the method. To further develop and validate this CT-free method using real patient data, we are currently conducting a clinical study (CASCTEC study 2022/04AOU/298). This study enrolls patients referred for myocardial perfusion scintigraphy, and includes both Std and Far acquisitions on the D530c system, with SPECT/CT serving as the reference standard. The results of this ongoing study should help to further evaluate the clinical applicability of the proposed approach, including the impact on clinical interpretation of discrepancies observed between the ground-truth image and the CT-free or CT-based AC image.

## Conclusions

This study introduced a novel patient-specific CT-free method for generating attenuation maps for cardiac-dedicated pinhole CZT cameras. These attenuation maps can be directly integrated into the manufacturer’s reconstruction workflow to produce AC cardiac images without requiring an external CT scan. The resulting AC images show activity recovery in good agreement with CT-based AC images acquired using the same camera and an external CT, and consistent with CZT SPECT/CT images, both in phantoms with and without myocardial perfusion defects.

These results demonstrate the potential of the proposed CT-free attenuation correction approach to substantially improve NAC images and, consequently, to enhance the diagnostic accuracy of myocardial perfusion scintigraphy performed with cardiac-dedicated pinhole CZT cameras. Further validation using patient data is required to confirm the clinical robustness of the method and to support its implementation in routine clinical practice.

## Supplementary Information

Below is the link to the electronic supplementary material.

Supplementary Material 1

## Data Availability

The datasets used and/or analyzed during the current study are available from the corresponding author on reasonable request.

## Abbreviations

AC: Attenuation Corrected
AHA: American Heart Association
AI: Artificial Intelligence
BMI: Body Mass Index
CT: Computed Tomography
CZT: Cadmium Zinc Telluride
DL: Deep Learning
FOV: Field of View
LV: Left Ventricular
MLEM: Maximum-Likelihood Expectation-Maximization
MP: Myocardial Perfusion
MRP: Median-Root Prior
NAC: Non-Attenuation Corrected
OSL: One Step Late
SPECT: Single Photon Emission Computed Tomography
