# Supplementary material for: Approximate CT-free patient specific attenuation correction method for cardiac-dedicated pinhole CZT system: a feasibility phantom study
Source: EJNMMI Phys. 2026 May 17;13:73. doi: 10.1186/s40658-026-00893-1 (PMC13346391; doi:10.1186/s40658-026-00893-1)
Supplement: Supplementary file 1 — Supplementary Material 1 [file 40658_2026_893_MOESM1_ESM.docx]

**Approximate CT-free patient specific attenuation correction method for cardiac-dedicated pinhole CZT system: a feasibility phantom study**

Michel Hesse PhD, Florian Dupont, Véronique Roelants MD, PhD

Nuclear medicine department, Cliniques Universitaires Saint-Luc, Brussels, Belgium

Corresponding author:

Michel Hesse

Service de médecine Nucléaire

Cliniques Universitaires Saint-Luc

10 avenue Hippocrate

1200 Brussels

B Belgium

Phone: +32 2 7642592

Fax: +32 2 7645408

Email: michel.hesse@uclouvain.be

**Short running title:** CT-free attenuation for cardiac SPECT

**Detailed description of the first step of the flowchart (Fig. 1) of the novel CT-free attenuation map generation method**

First the Far scan scatter projection data are converted into a body binary mask including all pixels exceeding 2% of the maximum pixel value across the 19 projections (Fig. S1a and S1b). To refine the mask boundaries, a one-pixel morphological opening operation is applied, followed by a hole-filling algorithm to eliminate any internal void.

Afterwards, a body volume is generated by including all voxels in the image space whose projections through each pinhole fall into the body binary mask (Fig. S1c). When the projection ends up beyond the CZT module associated to a pinhole, the closest body binary mask pixel value is used because only a part of the patient's body is seen by each pinhole (Fig. S1d). The body volume is generated within a volume of 44cmx44cmx44cm, larger than the standard D530c reconstruction volume (28cmx28cmx20cm centered on the heart), to ensure inclusion of all relevant tissues between the heart and the detectors. To refine the body volume boundaries, a morphological smoothing sequence is applied: 1 erosion, 3 dilations, and a final erosion using a kernel size of 3. The resulting volume is then used to generate a uniform attenuation map by assigning a fixed attenuation coefficient to all voxels.

***Fig. S1*** *CT-free uniform attenuation map generation*

**Figure S1** (a) Detector projections through the 19 pinholes for the TPN Far acquisition. (b) Body binary mask obtained by thresholding the Far scatter projections. (c) All voxels of the image space are projected through each pinhole (blue line) to check if they fall inside (pixel value at 1) or outside (pixel value at 0) the body binary mask. When the projection ends up beyond the CZT module (purple line), the value is set to the one of the closest detector pixel (red or green dotted lines). In the body volume, voxels whose projection falls inside (green squares) or outside (red squares) the mask are set to 1 and 0, respectively. (d) Body volume generated by combining the back-projections of the body mask through the 19 pinholes.

**Delineation of the heart on AC reconstructed image with a uniform attenuation map**

Figure S2 illustrates the steps for the determination of the smallest ellipsoid encompassing the heart in the reconstructed image of the Std scan with a uniform attenuation map. The gradient image with respect to the image center (Fig. S2b) is derived from the AC image that was trimmed to voxels seen by all pinholes (Fig. S2a). Then the gradient maxima are determined along all rays starting from the image center (which is close to the heart center for a correct heart positioning in the D530c FOV), and together they form an image of a rough border around the heart (Fig. S2c). This image is cleared by preserving only the largest connected structure in each frame (Fig. S2d). Finally, a mask is created by including only voxels located inside the border defined by the maximum gradient, eroding it twice to take into account that the gradient border is by design larger than the heart (Fig. S2e). This mask serves as input to the Khachiyan algorithm to produce the smallest ellipsoid encompassing the heart (Fig. S2f).

***Fig. S2*** *Generation of the smallest ellipsoid encompassing the heart*

**Figure S2** Steps to generate the smallest ellipsoid encompassing the heart. (a) Five slices including the heart in the reconstruction of the TP phantom with the uniform AC map. (b) Gradient of the image (a). (c) Image of the gradient maxima along rays originating from the image center. (d) Image (c) after removal of disconnected regions. (e) Mask defined by the voxels inside the gradient maxima border shown in (d). (f) Smallest ellipsoid (in red), that encompasses the heart, generated by the Khachiyan algorithm from the mask (e).

**Delineation of the left lung on reconstructed image of the scatter energy window**

The reconstructed image of the scatter energy window from the Std scan was first smoothed with a 3x3x3 voxels averaging filter (Fig. S3a), and will be referred as the scatter image in the following. The gradient of that image with respect to the heart ellipse center was generated, and a first approximation of the lung region was extracted as the volume between the minimal and maximal values of the gradient along rays originating from the ellipse center (Fig. S3b). In that first approximation the lung region was not allowed to expand beyond the heart center in the left-right direction to avoid including artefactual low counts voxels associated to regions not seen by all pinholes. In order to remove similar artefactual low count regions, typically located anterior to the heart, the lung region was segmented using the watershed method based on the scatter image (Fig. S3c). Only segments whose center falls below the heart ellipse center were kept, and the remaining region was smoothed to remove artefactual spikes (Fig. S3d). More specifically the smoothing was performed in each axial slice by voting in all pixels that have more than 4 pixels of their 8-neighbourhood inside the region, then voting out all pixels that have more than 4 pixels of their 8-neighbourhood outside the region. The lung region was then expanded in the posterior part of the body (Fig. S3e): for each axial slice, the mean value and standard deviation of voxels of the scatter image inside the lung region were computed. A threshold defined by the mean value plus one standard deviation was then used to expand the lung region to posterior voxels seen by at least 16 of the 19 pinholes. This final region defines the left lung in the axial slices that span the heart, and that are by design seen by all pinholes.

***Figure S3*** *Delineation of the lung region for the attenuation map*

**Figure S3** Steps to generate the lung region for the attenuation map. (a) The reconstructed image of the scatter energy window from the Std scan, after a 3x3x3 voxels averaging filtering. (b) The first approximation of the lung region obtained from the gradient of image (a), where the gradient was calculated with respect to the heart ellipsoid center (see supplementary figure 1). (c) The watershed segmentation of the lung region. (d) The lung region after removing the artefactual low count regions located above the heart (anterior area), identified from the previous segmentation. (e) The lung region after expanding it to lower count voxels in the posterior area.

**Extension of the lung region beyond heart slices**

After having generated a lung region in the axial slices encompassing the heart, this region was progressively extended to the upper slices (toward the patient shoulders) by adjusting the lung area of the previous slice (Fig. S4). The border of the lung area in the axial slice was first segmented by grouping the voxels according to their border normal vector. The lung area was then expanded or contracted for all the voxels of a group according to the gradient difference between voxels inside and outside of the lung area (Fig. S4b). Expansion (contraction) was performed if more than 60% of the border voxels show a larger (smaller) gradient on the outside than on the inside, and no change otherwise. If a contraction occurred for some axial slice, no more expansion was allowed for following slices, to favor a smoothly varying lung region.

***Figure S4*** *Extension of the lung region beyond heart slices*

**Figure S4** Illustration of the lung region extension beyond heart slices, towards shoulders. (a) One slice of the reconstructed image of the scatter energy window acquisition with the associated lung ROI (in red). (b) On the next slice, the gradient of the image is evaluated on both sides of the lung ROI border. Orange and green lines correspond to the border sides where the image gradient is lower and larger, respectively. The lung region is then adapted by expanding or shrinking the ROI border according to the border normal, i.e. following the green arrows. When no border side present a larger gradient (see the upper left border), no expansion nor shrinkage is performed on the local border. (c) The blue line defines the final lung area (after expansion) for the slice of panel (b).

**Co-registration of CT-free attenuation map with Starguide CT**

For each phantom configuration, the D530c NAC reconstruction of the Std scan was semi-automatically registered to the StarGuide CT-based AC image (MIM Software, Cleveland, OH). Because the CT-free attenuation map is intrinsically aligned with the NAC reconstruction, and the StarGuide CT scan and CT-based AC image are co-registered, applying the same transformation aligns the CT-free attenuation map with the CT.

**Phantoms reconstructions with and without attenuation correction**

The reconstructions with and without attenuation corrections of the 5 phantom setups are displayed on Fig. S5 for both cameras. The AC images from the D530c camera were obtained using the registered CT of the corresponding phantom acquired on the Starguide. The comparison of NAC and CTAC images helps in the interpretation of the activity defects observed in the CTAC images. Indeed, some structures appearing in the reconstructed images may be linked to the specific camera geometry and reconstruction algorithm. These structures should be of course similar for NAC and CTAC images. The D530c NAC images clearly display activity defects in the antero-septal and antero-lateral segments. These defects will not be recovered by the attenuation correction as they are located in shallow heart regions (see the last line in Fig. S5). The D530c CTAC images clearly show that the reconstructed activity distribution is very dependent on the defects inserted in the TP phantom: the heart activity appears more homogeneous in the TPN phantom without defect than for TPW and TPW2 phantoms that include 2 defects. Similar structural defects are visible on the Starguide image in the antero-septal and antero-lateral regions but are less marked, probably due to the larger FOV of the Starguide camera compared to the D530c.

***Fig. S5*** NAC and CTAC reconstructions of the phantoms

**Figure S5** NAC and CTAC reconstructions of the phantoms acquired on the Starguide and D530c cameras

**Impact of the manual edition of the CT-free attenuation map on HTP phantoms reconstructions**

Figure S6 presents the polar maps associated to the reconstructions of the HTP phantom setups with different attenuation maps on the D530c camera. The CTAC images from the D530c camera were obtained using the registered CT of the corresponding phantom acquired on the StarGuide. The NAC, CTAC and CT-free AC with manual edition images are the same as those shown in Fig. 4 and Fig. S5. The reconstructions on the second row of Fig. S6 correspond of the CT-free attenuation map without the manual edition of the body contour (yellow parts in Fig. 3). The impact of the polyurethane parts of the HTP phantoms is clearly observed by comparing rows 2 and 3 of Fig. S6. These parts, missed by the automatic body contouring of the CT-free method because of the lack of tracer uptake, induce an underestimation of the attenuation, leading to a hypo-perfusion in the anterior regions of the polar map. A partial recovery of that hypo-perfusion is observed after manual edition of the phantom body contour (row 3 of Fig. S6). The impact on the polar map is larger for HTP than for HTPo, as expected from the size of the missing parts (yellow in Fig. 3).

***Fig. S6*** NAC and AC reconstructions of the HTP phantom setups, with and without manual edition of the attenuation map

**Figure S6** NAC and AC reconstructions of the HTP phantom setups acquired on the D530c camera. The AC reconstructions were obtained with the attenuation map extracted from the Starguide CT, or with the CT-free attenuation map with or without the manual edition of the phantom body contour.

**Three-dimensional comparison of SPECT AC reconstructions of the 5 phantoms**

Figures S7-S11 present the three-dimensional AC reconstructions of phantom TPN, TPW, TPW2, HTP and HTPO, respectively. SPECT images are displayed in counts and presented according to the cardiac axes: short axis (SA), vertical long axis (VLA), and horizontal long axis (HLA). As observed in the bullseyes plots (Fig. 4), fully obstructive cardiac defects are clearly visible on all images, whereas the partially obstructive defect in the TPW2 configuration is only faintly visible on the ground-truth and StarGuide images. Figures S7-S11 illustrate the close agreement between the CT-free AC and CT-based AC reconstructions of the D530c data.

***Fig. S7*** SPECT AC reconstructions of the TPN phantom setup

**Figure S7** SPECT AC reconstructions of the TPN phantom setup. The ground-truth image, the StarGuide CT-based AC, the D530c CT-free AC and the D530c CT-based AC reconstructions are displayed on lines 1 to 4, respectively. The Images are displayed in counts and oriented according to the cardiac axes: short axis (SA), vertical long axis (VLA), and horizontal long axis (HLA).

***Fig. S8*** SPECT AC reconstructions of the TPW phantom setup

**Figure S8** SPECT AC reconstructions of the TPW phantom setup. The ground-truth image, the StarGuide CT-based AC, the D530c CT-free AC and the D530c CT-based AC reconstructions are displayed on lines 1 to 4, respectively. The Images are displayed in counts and oriented according to the cardiac axes: short axis (SA), vertical long axis (VLA), and horizontal long axis (HLA). The two fully obstructive cardiac defects are identified by the green and red arrows.

***Fig. S9*** SPECT AC reconstructions of the TPW2 phantom setup

**Figure S9** SPECT AC reconstructions of the TPW2 phantom setup. The ground-truth image, the StarGuide CT-based AC, the D530c CT-free AC and the D530c CT-based AC reconstructions are displayed on lines 1 to 4, respectively. The Images are displayed in counts and oriented according to the cardiac axes: short axis (SA), vertical long axis (VLA), and horizontal long axis (HLA). The arrows identify the fully obstructive cardiac defect (in green) and the partially obstructive cardiac defect (in red).

***Fig. S10*** SPECT AC reconstructions of the HTP phantom setup

**Figure S10** SPECT AC reconstructions of the HTP phantom setup. The ground-truth image, the StarGuide CT-based AC, the D530c CT-free AC and the D530c CT-based AC reconstructions are displayed on lines 1 to 4, respectively. The Images are displayed in counts and oriented according to the cardiac axes: short axis (SA), vertical long axis (VLA), and horizontal long axis (HLA).

***Fig. S11*** SPECT AC reconstructions of the HTPo phantom setup

**Figure S11** SPECT AC reconstructions of the HTPo phantom setup. The ground-truth image, the StarGuide CT-based AC, the D530c CT-free AC and the D530c CT-based AC reconstructions are displayed on lines 1 to 4, respectively. The Images are displayed in counts and oriented according to the cardiac axes: short axis (SA), vertical long axis (VLA), and horizontal long axis (HLA).
